# *TLR5* rs5744174 gene polymorphism is associated with the virus etiology of infant bronchiolitis but not with post‐bronchiolitis asthma

**DOI:** 10.1002/hsr2.38

**Published:** 2018-05-24

**Authors:** Sari Törmänen, Johanna Teräsjärvi, Eero Lauhkonen, Merja Helminen, Petri Koponen, Matti Korppi, Kirsi Nuolivirta, Qiushui He

**Affiliations:** ^1^ Center for Child Health Research Tampere University and University Hospital Tampere Finland; ^2^ Department of Medical Microbiology and Immunology Turku University Turku Finland; ^3^ Department of Pediatrics Seinäjoki Central Hospital Seinäjoki Finland; ^4^ Department of Medical Microbiology Capital Medical University Beijing China

**Keywords:** innate immunity, respiratory syncytial virus, single nucleotide polymorphism, Toll‐like receptor 5

## Abstract

**Background and aim:**

Bronchiolitis is a leading cause of hospitalization in infants and is associated with a risk of subsequent asthma. The innate immunity genes, such as those encoding toll‐like receptors (TLRs), are likely to play a role in bronchiolitis and post‐bronchiolitis outcome. Thus far, only one study has considered TLR5 genes in respiratory syncytial virus (RSV) bronchiolitis. The aim of this study was to investigate the association of TLR5 gene polymorphism with virus etiology and severity of bronchiolitis, and with post‐bronchiolitis asthma.

**Methods:**

We recruited 164 infants (age < 6 months) hospitalized for bronchiolitis in this study and determined TLR5 rs5744174 (C > T) single nucleotide polymorphism, virus etiology and severity markers of bronchiolitis, and presence of post‐bronchiolitis asthma until age 11 to 13 years.

**Results:**

RSV was detected in 113 (68.9%), rhinovirus in 19 (11.6%), and some other virus in 20 (12.2%) cases. Non‐RSV etiology was more common among infants with the variant CT or TT genotype in the TLR5 rs5744174 gene than in those with the CC genotype (89.7% vs 71.7%, P = 0.03). TLR5 rs5744174 polymorphism was not associated with the need of supplementary oxygen or feeding support, with the length of hospital stay, or with post‐bronchiolitis asthma at any age.

**Conclusion:**

The TLR5 rs5744174 variant genotype may increase the susceptibility to bronchiolitis not caused by RSV.

## 1 INTRODUCTION

Asthma is a chronic inflammatory disease of the airways, usually presenting with the dominance of Th2‐type cytokines.^1^ Both genetic susceptibility and environmental factors, such as early‐life virus infections, contribute to the development of asthma. Still, it remains unclear which abnormalities in the innate immunity ‐based host defense, such as cytokine misproduction, are hereditary, and which develop later due to environmental stress factors, such as infections.^2^

Bronchiolitis is the most common lower respiratory tract infection requiring hospitalization in young children.^3^ Among the various respiratory viruses causing bronchiolitis, respiratory syncytial virus (RSV) is the single most important one, especially in the youngest children.^4^, ^5^ The clinical course of primary RSV infection is highly variable, and genetic variations in genes regulating the immune response certainly influence disease severity.^6^ Bronchiolitis in infancy is associated with subsequent wheezing in early childhood and asthma in later childhood.^7^, ^8^, ^9^ The link between bronchiolitis and subsequent asthma remains unclear, but it has been suggested that the causative virus modifies the immature immunity towards a Th2‐oriented cytokine profile.^10^

Toll‐like receptors (TLRs) are innate immune molecules that recognize conserved structures of microbial pathogens.^11^ They activate immediate and early mechanisms of innate host defense as well as initiate and orchestrate adaptive immune responses.^11^ Several single‐nucleotide polymorphisms (SNPs) within the *TLR* genes have been associated with altered susceptibility to infectious, inflammatory, and allergic diseases.^11^, ^12^

TLRs 1, 2, 4, 5, 6, and 10 are located on the cell surface, directly recognizing microbial components, whereas TLRs 3, 7, 8, and 9 are located inside the cell,^13^ recognizing microbial components after endocytosis. TLR5 recognizes bacterial flagellin, which is an important virulence factor of many bacteria^14^ and is also found in house dust.^15^ The effect of bacterial products in house dust is mainly protective for allergy and asthma.^16^ In prior studies, activation of TLR5 by flagellin has been associated with atopic eczema^17^ and also with asthma,^15^ but there are studies with contradictory results reporting that asthmatic patients have decreased expression^18^, ^19^ or impaired function of TLR5.^19^ Moreover, polymorphisms in the *TLR5* gene have been described to be associated with both acute and chronic lung diseases, eg, pneumonia caused by *Legionella pneumophila* 20 and bronchopulmonary dysplasia in preterm infants.^21^

TLR5, as a receptor of bacterial flagellin, is known to be involved in allergy development and subsequent asthma.^15^ A Dutch study found preliminary evidence that the association of *TLR5* rs5744174 (Phe616Leu) polymorphism with bronchiolitis risk may be divergent in preterm and term infants.^22^ We have previously investigated the associations of SNPs of 9 *TLR* genes, not including the *TLR5* gene, with bronchiolitis and post‐bronchiolitis outcome.^23^, ^24^, ^25^, ^26^, ^27^ The aim of this study was to complete our previous exploratory studies by evaluating the association of *TLR5* rs5744174 gene polymorphism with bronchiolitis in infancy, with post‐bronchiolitis wheezing until the age of 18 months, with preschool asthma at the age of 5 to 7 years, and with childhood asthma at the age of 11 to 13 years.

## 2 MATERIALS AND METHODS

### 2.1 Design

The study was conducted at the Department of Pediatrics, Tampere University Hospital, Finland, and the design has previously been described.^9^ In brief, 187 previously healthy, full‐term infants hospitalized for bronchiolitis at less than 6 months of age in 2001 to 2004 were eligible for the study. Of them, 139 attended a follow‐up visit in 2002 to 2004 when the mean age of the children was 18 months. The second follow‐up visit was arranged in 2008 to 2010, at 5 to 7 years of age, to which 166 attended, and the third took place in 2014 to 2015, at the age of 11 to 13 years, to which 138 attended. Whole blood samples were obtained for genetic studies in infancy, supplemented at age 5 to 6 years, if needed. Overall, data on the *TLR5* rs5744174 (C > T) gene polymorphism were available from 164 infants. In infancy, bronchiolitis was defined as an acute lower respiratory illness characterized by rhinorrhea, cough, and diffuse wheezes or crackles.^28^ The viral etiology of bronchiolitis was studied by antigen detection and polymerase chain reaction (PCR) in nasopharyngeal aspirates, as described previously.^29^ The studied viruses were RSV, rhinovirus, human metapneumovirus, influenza A and B virus, parainfluenza type 1, 2, and 3 viruses, bocavirus, and adenovirus. Data on disease severity, such as the need for supplementary oxygen and feeding support, and the length of hospital stay (LOS), were recorded during the inpatient care.^27^

After hospitalization for bronchiolitis, the children were invited to a follow‐up visit at, on average, 1.5 years of age. At the follow‐up visit, the parents were interviewed using a structured questionnaire on the occurrence of atopic eczema, wheezing episodes, and the use of corticosteroids for wheezing after hospitalization for bronchiolitis.^30^ The parents had recorded all infections and wheezing periods diagnosed by a family doctor or a pediatrician and all given treatments in a diary during the 1.5 years post‐bronchiolitis follow‐up period. Only doctor‐diagnosed episodes were included in the analyses. Repeated wheezing was defined as 2 or more wheezing episodes during the 1.5‐year post‐bronchiolitis follow‐up period.^30^

The follow‐up study when the children were 5 to 7 years old, consisted of medical histories since the last visit at the age of 1.5 years. The data were collected using structured questionnaires, which the parents had completed before the visit, and a clinical examination by a doctor that included an interview, checking the questionnaire data, and an exercise challenge test with impulse oscillometry for bronchial hyper‐reactivity.^9^ Current asthma was defined as the current use of continuous maintenance medication with inhaled corticosteroids (ICS) for asthma, or suffering from doctor‐diagnosed episodes of wheezing after the age of 1.5 years, or from prolonged or night cough during the preceding 12 months, with a diagnostic finding in the exercise challenge test with impulse oscillometry.^9^ Previous asthma before the control visit was defined as the previous use of ICSs as continuous or intermittent maintenance medication for asthma.^9^

The follow‐up study, when the children were 11 to 13 years old, consisted of medical histories since the last visit at the age of 5 to 7 years. As in previous visits, data were collected using structured questionnaires, which the parents had completed before the visit, and a clinical examination by a doctor that included an interview and checking the questionnaire data. Lung function was measured with flow‐volume spirometer Vmax™ Carefusion (Becton, Dickinson and Company, NJ, USA), and a bronchodilation test was performed. Forced expiratory volume in 1 second (FEV1) was measured before and 15 minutes after salbutamol inhalation (Ventolin Evohaler 0.1 mg/dos, GlaxoSmithKline, London, UK) with a spacer and in both times, the best value out of 3 technically acceptable measurements was considered into the analyses. An increase of 12% in FEV1 was considered as diagnostic for asthma. Current asthma was considered to be present if the child had used ICSs continuously during the last 12 months. It was also considered to be present if the child had suffered from repeated wheezing or from a prolonged cough or night cough for 4 or more weeks during the last 12 months, and, in addition, had a diagnostic increase of FEV1 in the bronchodilation test. Persistent asthma was considered to be present if the child with current asthma had also had asthma at the last control visit at the age of 5 to 7 years.

At both the 5 to 7‐year and the 11 to 13‐year visit, the questionnaire consisted of questions concerning doctor‐diagnosed atopic eczema, allergic rhinitis, and food allergies at different ages since the last control visit. It also covered the current use of asthma medication, current symptoms that could suggest asthma, and the current asthma diagnosis, if present.

### 2.2 Genetic studies

High‐resolution melting analysis (HRMA) (Roche Diagnostics Light Cycler 480, Basel, Switzerland) was used for genotyping of *TLR5* rs5744174 (C > T) polymorphism, as published recently.^31^HRMA is a post‐PCR melt analysis method based on the detection of changes in fluorescence due to the binding of a double stranded‐specific intercalacting fluorescent dye, at different temperatures. Primers (forward 5´‐ACCTTCCGTGGAAAGAGAGAA‐3′ and reverse 5´‐TGCAGACATATATTGTGTGTACCCT‐3) were designed with Primer‐Blast design tool. Amplicon size was only 70 bp which is small enough to maximize the difference between melting peaks (T_m_) in variant genotypes and to avoid the other SNPs.^32^ Three samples with known genotypes were used to determine the proper concentration of MgCl_2_ and annealing temperature (T_a_°C) for assay.

Each run reaction (20 μL total) consisted of 3‐μL genomic DNA (~8.0 ng/μL) and 17 μL of master mix, which includes 10‐μL melting master dye (LightCycler 480 High Resolution Melting Master, Product No.04909631001, Roche, Basel, Switzerland), 2.4 μL of MgCl_2_ with a final concentration of 3 mM, 1 μL of each primer with a final concentration of 0.2 μM, and 2.6 μL of water. The Master mix provides a final concentration of 3 mM of MgCl_2_. HRMA reactions were run at 95°C for 10 minutes followed by 45 cycles amplification at 95°C for 10 seconds, at 60°C for 10 seconds and at 72°C for 15 seconds. After the PCR run, final melting cycle conditions were as outlined by Roche: first heating to 95°C and hold for 1 minute and cooling to pre‐hold temperature (40°C). Melting interval for collecting fluorescence from 60°C to 95°C at ramp rate 0.02°C per second. In each run, known *TLR5* rs5744174 standards (wild type, heterozygote, and homozygote) were used.

The 1000 Genome Project FIN data on *TLR5* rs5744174 gene polymorphism was obtained from 99 Finnish subjects,^33^ and the minor allele frequencies (MAF) were compared between our cases and that FIN data.

### 2.3 Ethics

The study was carried out in accordance with the approved guidelines of the WMA Declaration of Helsinki. The protocol of the study was approved by the Ethics Committee of the Tampere University Hospital District, Tampere, Finland. Before enrolling the children, we obtained informed parental consent, including the use of samples for genetic studies on bronchiolitis and asthma risk, both during hospitalization and at the control visit. The personal data of the study subjects were not given to the laboratory that performed the genetic studies, the Department of Medical Microbiology and Immunology, University of Turku, Finland.

### 2.4 Statistics

Statistical analyses were carried out with SPSS version 23.0 software (IBM Corp, NY, USA). The Chi‐square test and Fisher's exact test were used, as appropriate, for categorized variables. Logistic regression with adjustments for sex and age was used to analyze the association between the *TLR5* rs5744174 genotypes and virus etiology of bronchiolitis. The results were expressed as odds ratios (OR) and 95% confidence intervals (95% CI).

The FINETTI program, version 3.0.5 (http://ihg.gsf.de/cgi-bin/hw/hwa1.pl) was used to evaluate the Hardy‐Weinberg equilibrium of the studied *TLR5* alleles, and they were in the Hardy‐Weinberg equilibrium.

## 3 RESULTS

### 3.1 Hospitalization data

There were 164 (87.7%) patients with available genetic and clinical data during hospitalization. Eighty‐three (50.6%) of them were boys. Fifty‐seven (34.8%) children needed feeding support, and 31 (18.9%), oxygen supplementation during the hospitalization. The mean age of the children was 10.6 weeks (range 1‐25 weeks, SD 6.77). The mean LOS in hospital was 4.49 days (range 0‐22 days, SD 3.21). The causative virus was RSV in 113 (68.9%) cases (RSV A in 61 and RSV B in 52 cases), rhinovirus in 19 (11.6%), and some other virus in 20 (12.2%) (human metapneumovirus in 6, Influenza A virus in 9, parainfluenza type‐3 virus in 4 and adenovirus in 1 case). Thus, non‐RSV etiology of bronchiolitis was present in 39 (23.8%) cases. The virus was not identified in 12 (7.3%) cases.

The *TLR5* rs5744174 genotype was CC in 39 (23.8%) cases, variant CT in 84 (51.2%) cases, and variant TT in 41 (25%) cases. There was no significant association between sex and the genotypes. Variant genotypes (CT or TT) were more common in infants with bronchiolitis caused by some other virus (non‐RSV group) than by RSV (89.7% vs 71.7%, *P* = 0.03) (Table 1.). The OR was 3.46 (95% CI 1.14‐10.52), and when adjusted by age and sex, the aOR was 3.17 (95% CI 1.03‐9.74). When RSV A and RSV B were included separately in supplementary analyses, the difference was still clear. The variant genotypes (CT or TT) were more common in infants with bronchiolitis caused by non‐RSV (89.7%) than by RSV A (70.5%, *P* = 0.02) or by non‐RSV than by RSV B (73.1%, *P* = 0.04). Concerning RSV A, the OR was 3.66 (95% CI 1.14‐11.82) and the aOR was 3.22 (95% CI 0.98‐10.62), and concerning RSV B, the OR was 3.22 (95% CI 0.97‐10.73) and the aOR 3.01 (95% CI 0.90‐10.13).

**Table 1:** *TLR5* rs5744174 genotypes and minor allele T frequencies in relation to virus etiology of bronchiolitis calculated by Chi‐square test or Fisher's exact test

	RSV *n* = 113	Non‐RSV *n* = 39		Rhinovirus *n* = 19		Virus other than RSV or Rhinovirus *n* = 20	
Genotype	*n* (%)	*n* (%)	*P* *	*n* (%)	*P* *	*n* (%)	*P* *
CC *n* = 39	32 (28.3)	4 (10.3)	‐‐‐	2 (10.5)	‐‐‐	2 (10.0)	‐‐‐
CT (variant) *n* = 84	57 (50.4)	20 (51.3)	0.08	10 (52.6)	0.33	10 (50.0)	0.33
TT (variant) *n* = 41	24 (21.2)	15 (38.4)	0.008	7 (36.8)	0.07	8 (40.0)	**0.04**
CT or TT (variant) *n* = 125	81 (71.7)	35 (89.7)	**0.03**	17 (89.5)	0.15	18 (90.0)	0.10
Minor allele T frequency *n* = 164/328	105/226 (46.5) *P* = 0.47 vs the FIN data **	50/78 (64.1) ***P* = 0.03** vs the FIN data		24/38 (63.2) *P* = 0.14 vs the FIN data		26/40 (65.0) *P* = 0.08 vs the FIN data	

The significant *p*‐values are expressed in bold.

* vs the genotype CC.

** FIN data from the 1000 genomes project.^33^

There were no significant associations between the *TLR5* rs5744174 genotypes and the severity markers of bronchiolitis, such as the need of feeding support, oxygen supplementation, or the LOS (Table 2.).

**Table 2:** *TLR5* rs5744174 genotypes in relation to the severity markers of bronchiolitis, including the need of feeding support or oxygen supplementation during hospitalization, and the length of hospital stay, calculated by Chi‐square test or Fisher's exact test

	Genotype CC *n* = 39		Genotype CT or TT (variant) *n* = 125		
Clinical finding	*n*	%	*n*	%	*P*‐value
Feeding support *n* = 57	13	33.3	44	35.2	0.83
Oxygen supplementation *n* = 31	78	17.9	24	19.2	0.86
Length of hospital stay (mean, SD)	5.21 (SD 3.88)		4.27 (SD 2.95)		0.11

### 3.2 Comparison with FIN data

The MAF (allele T) in this study population was 0.51 and did not differ from the general Finnish population according to FIN data of the 1000 Genomes Project,^33^ where the MAF of the *TLR5* rs5744174 (C > T) was 0.50. In the RSV group, the MAF was 0.47 (*P* = 0.47 vs the FIN data), and in the non‐RSV group, the MAF was significantly higher, at 0.64 (*P* = 0.03 vs the FIN data) (Table 1).

### 3.3 1.5‐year follow‐up visit

There were 112 children with clinical data available from the 1.5‐year follow‐up visit. The *TLR5* rs5744174 genotype was CC in 22 (19.6%) cases and CT or TT (variant) in 90 (80.4%) cases. There were no significant associations between the *TLR5* rs5744174 genotype and post‐bronchiolitis wheezing, presence of atopic eczema, or the use of ICS medication (Table 3).

**Table 3:** *TLR5* rs5744174 genotypes in relation to clinical outcome at 1.5 years of age after bronchiolitis in infancy, calculated by Chi‐square test or Fisher's exact test

	Wild Genotype (CC) *n* = 22		Variant Genotype (CT or TT) *n* = 90		
Clinical finding	*n*	%	*n*	%	*P*‐value
Atopic eczema *n* = 15	1	4.5	14	15.6	0.16
Repeated wheezing *n* = 21	4	18.2	17	18.9	0.60
ICS use *n* = 16	3	13.6	13	14.4	0.59

### 3.4 5 to 7‐year follow‐up visit

There were 139 children with clinical data available from the 5 to 7‐year follow‐up visit. The *TLR5* rs5744174 genotype was CC in 36 (25.9%) cases and CT or TT (variant) in 103 (74.1%) cases. There were no significant associations between the *TLR5* rs5744174 genotype and prolonged cough, ICS use, current asthma, or presence of atopic eczema or allergic rhinitis (Table 4).

**Table 4:** *TLR5* rs5744174 genotypes in relation to clinical outcome at 5 to 7 years of age after bronchiolitis in infancy, calculated by Chi‐square test or Fisher's exact test

	Wild Genotype (CC) *n* = 36		Variant Genotype (CT or TT) *n* = 103		
Clinical finding	*n*	%	*n*	%	*P*‐value
ICS use *n* = 17	7	19.4	10	9.7	0.13
Current asthma *n* = 15	6	16.7	9	8.7	0.19
Allergic rhinitis *n* = 40	13	36.1	27	26.2	0.26
Atopic eczema *n* = 40	8	22.2	32	31.2	0.31

### 3.5 11 to 13‐year follow‐up visit

There were 123 children with clinical data available from the 11 to 13‐year follow‐up visit. The *TLR5* rs5744174 genotype was CC in 32 (26.0%) cases and CT or TT (variant) in 91 (74.0%) cases. There were no significant associations between the *TLR5* rs5744174 genotypes and prolonged cough, ICS use, current asthma, persistent asthma continuing from preschool age until the latest follow‐up visit, or presence of atopic eczema or allergic rhinitis (Table 5.).

**Table 5:** *TLR5* rs5744174 genotypes in relation to clinical outcome at 11 to 13 years of age after bronchiolitis in infancy, calculated by Chi‐square test or Fisher's exact test

	Wild Genotype (CC) *n* = 32		Variant Genotype (CT or TT) *n* = 91		
Clinical finding	*n*	%	*n*	%	*P*‐value
ICS use *n* = 10	3	9.4	7	7.7	0.72
Current asthma *n* = 14	6	18.8	8	8.8	0.13
Persistent asthma *n* = 8	3	9.4	5	5.5	0.43
Allergic rhinitis *n* = 53	11	34.4	42	46.2	0.25

## 4 DISCUSSION

There are 3 main results in this study concerning the *TLR5* rs5744174 (C > T) gene polymorphism in bronchiolitis and post‐bronchiolitis outcome. First, the *TLR5* rs5744174 variant genotype was associated with non‐RSV etiology of bronchiolitis. Second, *TLR5* rs5744174 polymorphism was not associated with severity of bronchiolitis. Third, *TLR5* rs5744174 polymorphism was not associated with post‐bronchiolitis wheezing, preschool asthma, or current asthma in 11 to 13‐year‐old children after bronchiolitis in infancy.

Approximately 2% to 3% of children are hospitalized for bronchiolitis before the age of 12 months.^34^ In a recent Finnish study, the figure was 2.6% in infants under 6 months of age.^35^ RSV is the most common cause for bronchiolitis, especially among infants less than 12 months, whereas other viruses, especially rhinovirus, become more frequent after that age.^4^ Consistent with this, RSV caused 68.9% of bronchiolitis cases (74.3% of the virus‐positive cases) in the present study in infants hospitalized for bronchiolitis at less than 6 months of age. However, bronchiolitis caused by some other virus than RSV was significantly more common among children with the variant genotype in the *TLR5* rs5744174 gene. This result was confirmed with adjusted analyses when infants with non‐RSV bronchiolitis were compared with all RSV cases, but the significance was marginally lost when compared with RSV A and RSV B cases separately, although the risk was more than 3‐fold in all analyses. Further, the MAF was significantly higher in the non‐RSV group (0.64) than in the Finnish population (0.50), according to the FIN data of the 1000 Genome Project.^33^

There is some evidence that polymorphisms in the *TLR5* gene are associated with bacterial infections,^20^, ^36^ but their role in viral infections is less studied. So far, there has been only one study on TLR5 in infant bronchiolitis, where *TLR5* rs5744174, the same SNP as in the present study, did not have any significant effect on the risk of RSV bronchiolitis.^22^ In stratified analyses, however, preliminary evidence was found that the influence may be divergent in term and preterm infants with bronchiolitis. No studies are available on the association between *TLR5* genes and bronchiolitis caused by other viruses, or between *TLR5* genes and the post‐bronchiolitis outcome.

The *TLR5* rs5744174 site is located in the coding region of the gene, and the SNP results in a missense mutation, where the 616^th^ amino acid of the TLR5 protein, phenylalanine (Phe), is substituted by a leucine (Leu).^37^ Depending on its location, polymorphisms in the encoding gene of TLRs may affect the level of expression or alternatively, lead to an altered binding affinity or to an altered downstream signaling.^38^ Subsequently, these changes in the primary defense may lead to attenuated immune responses and, further, to increased susceptibility to infections. In primary human cell cultures, *TLR5* rs5744174 polymorphism was found to attenuate TLR5 signaling in response to bacterial flagellin.^39^ In addition to recognizing flagellin, there is preliminary evidence that TLR5 might have immune modulating effects in virus infections. A recent study found that flagellin‐induced activation of TLR5 prevented rotavirus infection in mice through activating innate immunity.^40^ Moreover, flagellin has been shown to be effective as an adjuvant in influenza vaccines by triggering TLR5 activity and boosting immune responses.^41^, ^42^ In the present study, the *TLR5* rs5744174 variant genotype was associated with an increase in susceptibility to non‐RSV bronchiolitis, which may take place via an attenuated function of TLR5.

The data on the role of TLR5 in asthma is inconsistent, and even less is known about the effect of the genetic variants of the *TLR5* rs5744174 gene on asthma susceptibility. In the present study, *TLR5* rs5744174 polymorphism had no significant association with recurrent wheezing or asthma during the longer than 10 years follow‐up after bronchiolitis in infancy. This is in agreement with a study of a German population in which no significant associations between *TLR5* rs5744174 polymorphism (the polymorphism studied here), or 2 other *TLR5* gene polymorphisms, rs5744168 and rs2072493, and childhood asthma, were found.^38^

It has been suggested, that in healthy subjects, the activation of TLR5 induces Th1‐type immune responses, but in asthmatic patients, the expression and activation of TLR5 and subsequent release of Th1‐type and anti‐inflammatory cytokines are decreased, and the normal function of TLR5 probably is protective from asthma.^19^ In another study, TLR5 expression was decreased only in severe asthmatics.^18^ A different study reported contradictory results, proposing that the activation of TLR5 leads to Th2‐oriented responses in mice.^43^ Further, a few studies have implicated that the effect of contact with bacterial flagellin may be dose dependent: a small amount of flagellin may promote asthma by priming allergic responses,^15^ whereas higher amounts may suppress allergic asthma.^44^

Compared with other viruses, RSV is associated with more severe symptoms of bronchiolitis, whereas rhinovirus is more commonly associated with post‐bronchiolitis asthma than is RSV.^45^ In the present study, *TLR5* rs5744174 genotypes did not associate with severity markers of bronchiolitis during hospitalization, although non‐RSV bronchiolitis was more common among children with variant genotypes of the *TLR5* rs5744174 gene. This may be due to the small sample size, as only 39 children had bronchiolitis caused by some other virus than RSV. It is also notable, that a loss‐of‐function mutation in the ligand‐binding domain of *TLR5*, 392STOP, was associated with defective responses to flagellin and increased susceptibility to Legionnaire's disease.^20^ This polymorphism is present in 5% to 10% of Europeans and in 23% of some other populations, and when present in both alleles, leads to a complete TLR5 defect without any general immunodeficiency.^46^ Thus, TLR5 seems not to be crucial for host defense, and in case of attenuated or even complete loss of function, other molecules of innate immunity are able to compensate for its lacking function.^46^

The strengths of this study are the prospective design and carefully collected data during both the hospitalization and repeated follow‐up visits, and a long follow‐up time of over 10 years. The homogeneous ethnic background of the study children is also a benefit in genetic studies. One clear limitation of this study, however, is the fact that we were not able to measure the expression of TLR5 or the functionality of the *TLR5* rs5744174 gene. However, the functionality of this polymorphism has been proven in previous studies.^39^ The small sample size is a limitation for genetic studies and, therefore, the findings need to be repeated in larger study populations.

In conclusion, the *TLR5* rs5744174 variant genotypes were associated with non‐RSV etiology of bronchiolitis, but not with the severity of bronchiolitis or with the incidence of post‐bronchiolitis asthma. Thus, the findings of this exploratory study suggest that TLR5 may have a modulating role in virus bronchiolitis but larger confirmatory studies in other populations are needed.

## CONFLICTS OF INTEREST

None declared.

## AUTHOR CONTRIBUTIONS

Conceptualization: Matti Korppi, Kirsi Nuolivirta
Data Curation: Sari Törmänen, Kirsi Nuolivirta, Eero Lauhkonen, Petri Koponen
Formal Analysis: Sari Törmänen, Kirsi Nuolivirta
Investigation: Sari Törmänen, Eero Lauhkonen, Kirsi Nuolivirta, Petri Koponen, Merja Helminen, Johanna Teräsjärvi, Qiushui He
Methodology: Matti Korppi, Kirsi Nuolivirta, Merja Helminen, Qiushui He
Project Administration: Matti Korppi, Kirsi Nuolivirta
Resources: Matti Korppi, Kirsi Nuolivirta, Merja Helminen, Qiushui He
Supervision: Matti Korppi, Kirsi Nuolivirta
Writing—Original Draft Preparation: Sari Törmänen, Johanna Teräsjärvi, Matti Korppi, Kirsi Nuolivirta, Qiushui He
Writing—Review and Editing: Eero Lauhkonen, Merja Helminen, Petri Koponen
